# A minimally invasive catheterization of the external jugular vein in suckling piglets using ultrasound guidance

**DOI:** 10.1371/journal.pone.0241444

**Published:** 2020-10-28

**Authors:** Hauteclaire Furbeyre, Etienne Labussiere

**Affiliations:** PEGASE, INRAE, Institut Agro, Saint-Gilles, France; University of Illinois, UNITED STATES

## Abstract

There is a growing interest for minimally invasive surgical procedures to improve experimental animal welfare. Minimally invasive catheterization procedures in pigs have been already developed using Seldinger technique but reproducibility is low, especially in young pigs. A novel method for a minimally invasive catheterization of external jugular vein was evaluated in suckling piglets of 21 days of age. Growth performance and haptoglobin concentration in plasma were measured throughout a four-week study in a group of seven catheterized piglets and a group of seven non-catheterized piglets. Catheterization was performed using Seldinger technique under continuous ultrasound monitoring for vein detection and needle insertion. The surgical procedure was quick and showed a great reproducibility. All catheters remained functional during the first week after catheterization. Catheterization in piglets did not significantly affect body weight (BW) and feed intake during four weeks after the surgical intervention compared to non-catheterized piglets (*P* > 0.10). Haptoglobin concentration in plasma was greater in catheterized piglets compared with non-catheterized piglets, with a significant increase over two weeks after catheter insertion (*P* < 0.05), suggesting the development of a chronic inflammation in catheterized piglets. This method can be easily performed in piglets with minimal effect on growth and feeding behaviour. Transposition to heavier pigs should be considered.

## Introduction

Venous catheterization is often performed in pigs for biomedical or nutritional research. Venous catheters allow repetitive blood samplings or intravenous administration of drugs or metabolic markers with minimal contention or stress for animals. In pigs, minimally invasive catheterization can be performed in peripheral veins such as auricular vein, but are not suitable for long-term experiments [1]. Consequently, long-term experiments often refer to invasive surgical procedures to get access to the central venous system [1]. The latter needs a relatively long time for recovery, the use of perioperative drugs to prevent pain and inflammation caused by tissue trauma, and most of the time antibiotic administration to prevent from bacterial contamination and septicaemia. There is a growing interest for minimally invasive surgical procedures to 1. improve experimental animal welfare and 2. reduce biases induced by surgery through drug administration or animal behaviour changes. Some authors evaluated percutaneous catheterization in young pigs using the Seldinger technique [2, 3]. Percutaneously inserted catheter minimises inflammation and tissue damage and prevents heavy peri-operative treatments. However, blind puncture of the external jugular vein in very young pigs may lead to multiple tries before success or cause inadvertent arterial puncture [2–4]. Ultrasound guidance is a useful tool to help identify venous and arterial vessels and may contribute to a quick and reproducible venepuncture of the external jugular vein in piglets [5]. The aim of this study was to develop a simple and reproducible procedure for external jugular vein catheterization in suckling piglets weighing seven kilograms using ultrasound guidance with minimal impact on piglet feeding behaviour, growth performance and health status.

## Materials and methods

### Animals

This experiment was conducted in the experimental facilities of INRAE. This experiment was approved by the Committee on the Ethics of Animal Experiments of Rennes (Comité Rennais d’Ethique en matière d’Expérimentation Animale), registered in the French National Committee of Ethic Reflexion for Animal Experiments (authorization no. 2017102109509867) under the national ethical guidelines for use of animals for scientific purposes (Ordinance of 2013 February 1st relative to the ethical assessment of animal use in experimental procedures) in accordance with the European Convention for the Protection of Vertebrate Animals used for Experimental and Other Scientific Purposes. The experiment was conducted with 14 suckling castrated male piglets of 21 days of age (initial BW = 7.3 ± 0.6 kg) selected as pairs from the same litter. Only castrated male piglets were used in this study to avoid any sex effect on growth performance and inflammatory status. Crossbred piglets (Pietrain x (Large White x Landrace)) were selected from the experimental herd of INRAE Saint Gilles. Within pairs, piglets were either allocated to the Control group (*n* = 7) or the Catheter group (*n* = 7). Groups were built with specific attention to have a similar and homogenous initial BW in the two groups. In the Catheter group, a catheter (Leadercath 2 E.L., Vygon, Ecouen, France) was inserted in the left external jugular vein at 21 days of age (d0) using percutaneous catheterization. Piglets returned with their littermates under their mother at the end of the surgical procedure. Piglets from the Catheter and Control group were raised under the sow for 7 days (d0 to d7). Piglets from the Control group did not receive any particular treatment from d0 to d7. All piglets were then weaned on d7 in individual cages equipped with a slatted floor with *ad libitum* access to feed and water until d28. Piglets were weighed at d0, d7, d14, d21 and d28. Feed was sampled daily for dry matter (DM) determination after drying at 103°C during 24 hours. Feed refusals were collected daily and dried at 103°C for 24 hours. Feed consumption was then measured daily on a DM basis as the difference between offered DM and refused DM. Catheterized piglets were euthanized with a lethal dose of T61 (60 mg embutramide, 8.08 mg mebezonium iodide, 1.32 mg tetracaine hydrochloride per kg BW, MSD Santé Animale, Beaucouzé, France) injected through the catheter. Piglets were necropsied at the end of the experiment at d28.

### Catheter insertion

**Fig 1:**
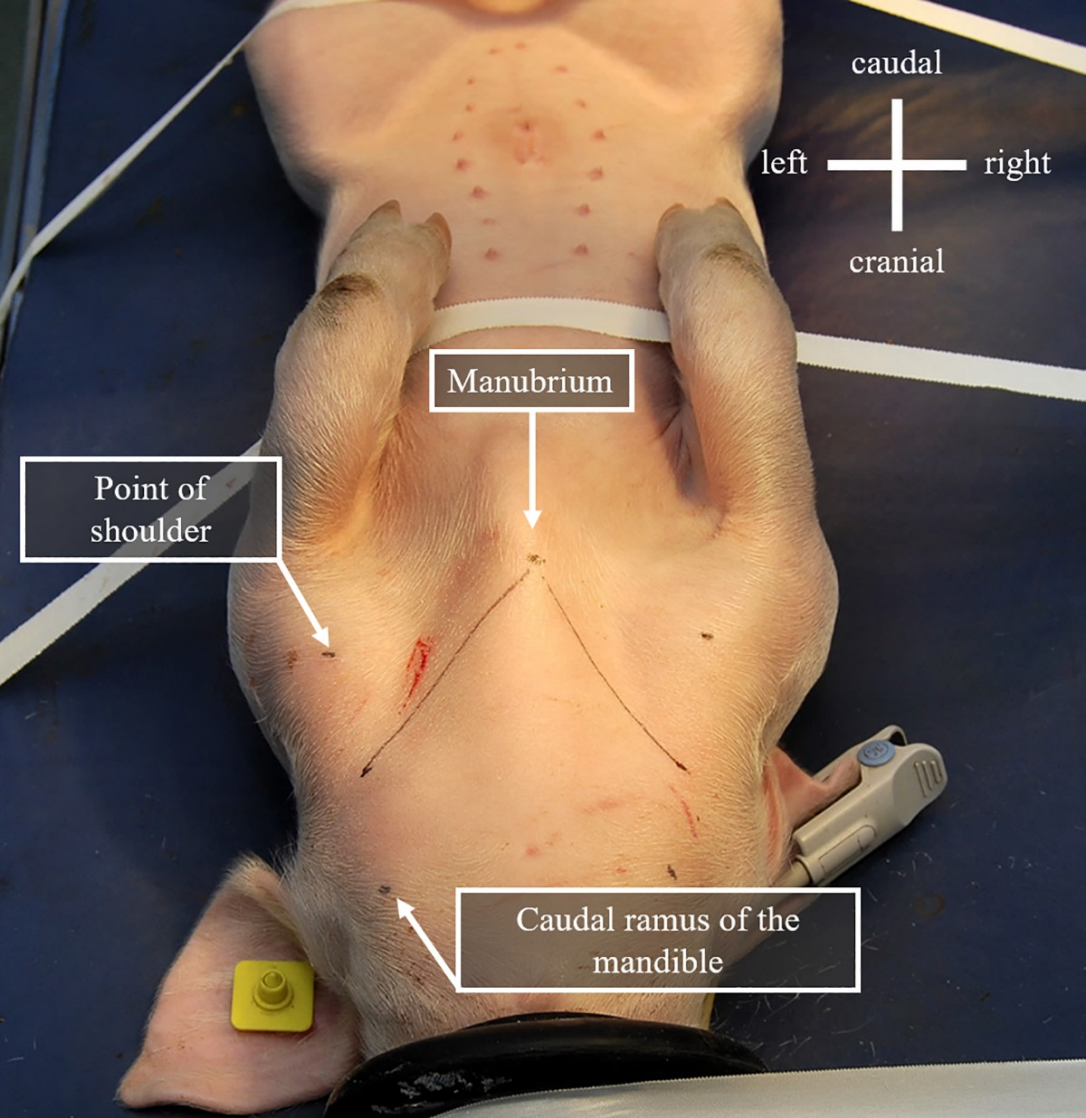
Landmarks for the determination of the expected passage of the external jugular vein in 21 days old piglet.

Piglets were moved individually from the maternity pen to the surgical theatre using a closed box to prevent from thermal cold stress. Anaesthesia was induced by placing the piglet under a face mask delivering 5% isoflurane in 100% oxygen. After vigilance breakdown, isoflurane delivery was reduced to 2.5%. Pulse oximetry was monitored throughout the anaesthesia. Piglet was placed in dorsal recumbency. Hair was clipped using a surgical scalpel blade from the insertion site and the surrounding area approximately from the points of shoulders to the caudal ramus of mandibles (Fig 1).

Specific landmarks were determined to identify the external jugular vein passage and help orientating ultrasound guide. As described in Fig 1, three points were marked under full extension of the ipsilateral limbs: the cranial manubrium, the point of shoulder and the caudal ramus of the mandible. The expected passage of external jugular vein was drawn as the line running from the cranial manubrium to the point at the middle distance between the shoulder and the caudal ramus of the mandible, as shown in Fig 1. This line was used as the “way to follow” to detect the external jugular vein with the ultrasound transducer.

**Fig 2:**
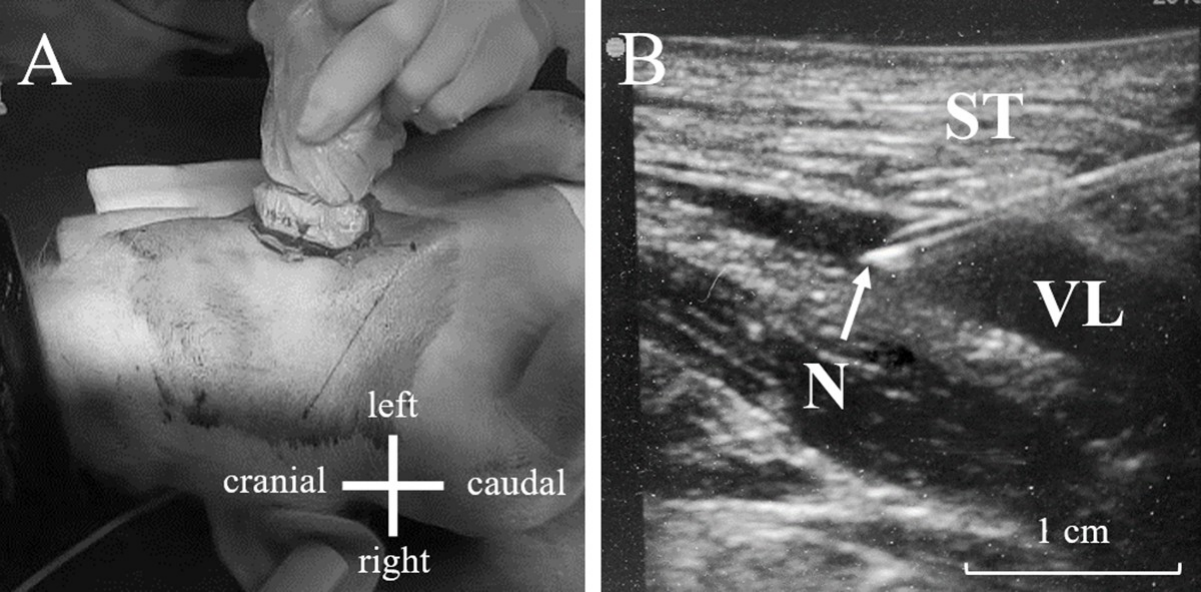
A. Photograph that shows the positioning of the ultrasound transducer on the marked line (expected passage of external jugular vein); B. Visualization of the 21G echogenic needle entering the venous lumen; ST = subcutaneous tissues (muscle); VL = venous lumen; N = echogenic needle.

The clipped zone was cleansed using polyvidone iodine solution (Vétédine® Solution; Vetoquinol, Paris, France). The ultrasound monitor (Sonosite M-Turbo®, Fujifilm Sonosite, Paris, France) was equipped with a 13–6 MHz transducer (Sonosite L25, Fujifilm Sonosite, Paris, France), covered with ultrasonic gel and wrapped in a sterile plastic sheath (CIV-FLEX™, EDM Medical Imaging, Domont, France). Ultrasonic gel was placed over piglet’s skin on the expected passage of the external jugular vein. The ultrasound transducer was then placed on or parallel to the line previously described in Fig 1. Catheterization was then performed under continuous observation of real-time 2D imaging recorder with the ultrasound monitor (Fig 2). Blood flux detection was performed using the “Color” function of the ultrasound monitor. The external jugular vein was localized 1 to 2 cm deep under the skin and vein circumference averaged about 0.5 cm diameter (Fig 2). External jugular vein catheterization was performed in two steps, using at first a commercial microintroducer set (V-Stick, Argon Medical, Frisco, USA) and then a 18G x 10 cm single lumen catheter kit (Leadercath 2 E.L., Vygon, Ecouen, France). After vein detection using ultrasound monitoring, a 21G echogenic needle was inserted through the skin with 15° angle onto the external jugular vein lumen. A 0.5 mm guidewire was then inserted into the needle. Guidewire positioning in the lumen of the vein was confirmed using ultrasound monitoring. A portion of the guidewire was kept into the lumen of the vein and the needle was removed. A 4-Fr rigid dilator was then advanced over the guidewire through the skin and subcutaneous tissues. The guidewire was removed and replaced by a 0.7 mm guidewire from the 18G catheter kit. The dilator was removed gently. The 18G catheter was guided into the vein with the guidewire before the latter being removed.

**Fig 3:**
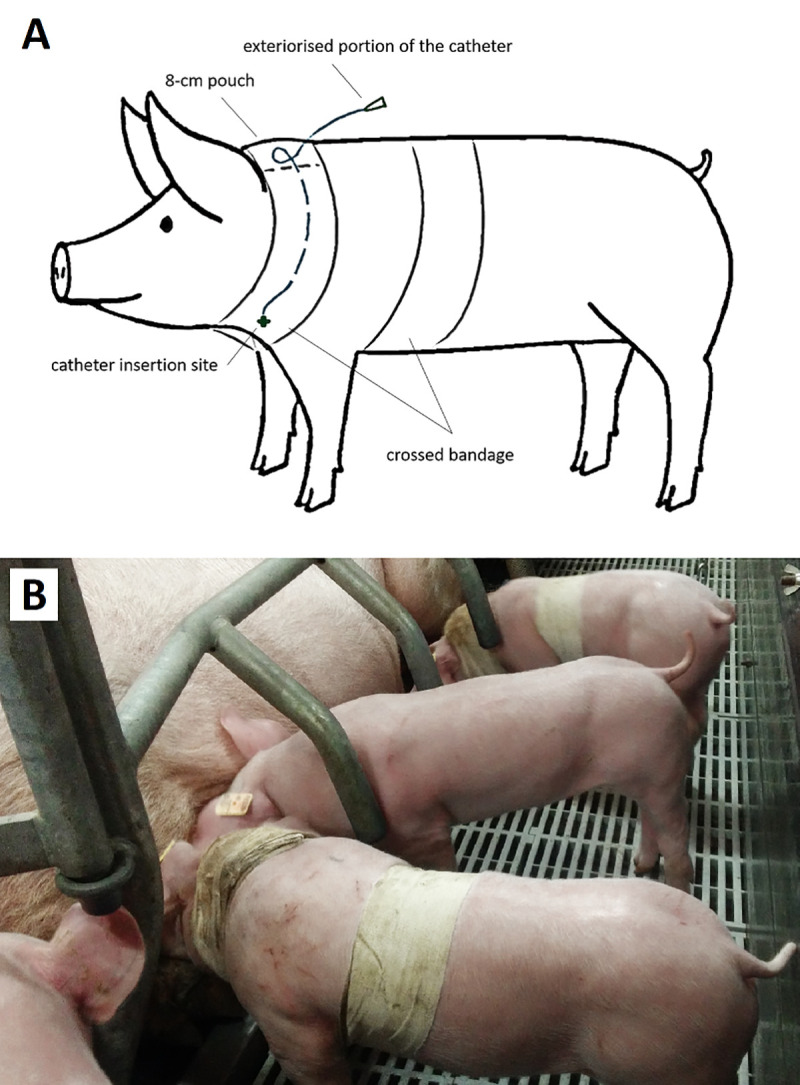
A. Schematic view of catheter positioning in 21-day-old piglets at the end of the catheterization procedure. B. Photograph that shows piglets equipped with catheter during the suckling period. The catheter was covered with a crossed bandage around piglet’s neck and abdomen to ensure overall protection of catheter insertion site. The catheter was routed between two layers of bandage and the exteriorised portion of catheter was placed in a 8×8-cm pouch on the dorsal surface of the neck. Piglets were group-housed for 7 days after the surgical procedure.

The catheter was flushed with saline solution (NaCl 0.9%) containing 1% heparin. Catheter was equipped with a 30-cm extension (Lectro-cath, Vygon, France) flushed with saline solution containing 1% heparin. The catheter was secured with one suture bound to the catheter basis. The catheter was finally covered with a crossed bandage around piglet’s neck and abdomen using an 8 cm width bandage (Tensoplast vet, BSN Medical, Leuven, Belgium). Catheter was placed in order to be accessible from the dorsal surface of the neck using the method of Matte [6]. Briefly, a catheter extension was routed to the dorsal surface of the neck between two layers of bandage and the exteriorised portion of catheter was placed in a 8×8-cm pouch (Fig 3A).

At the end of the surgical procedure, isoflurane anaesthesia was stopped and facial mask was removed. After anaesthesia removal, piglets were kept isolated from their littermates in a box until full recovery. Self-mobility and suckling recovery was checked for each piglet when it returned with its littermates (Fig 3B). Taken into account that anti-inflammatory and anti-microbial substances may interfere with health status around weaning, we would like to avoid the use of analgesic or prophylactic substance during and/or after the surgical procedure. For ethical concerns, heart rate was checked throughout the surgical procedure to detect variations that may be associated with pain. Post-operative exhibited signs of pain such as abnormal posture or gait, social isolation, vocalization, elevated respiratory rate and decreased appetite (expressed via isolation during suckling events within the seven days post-surgery) were evaluated for each pig within the first hour following the surgical procedure, then twice daily throughout the experiment. If a piglet exhibited one of the described signs of pain, it would be excluded of the experiment to receive an intramuscular administration of meloxicam (0.4mg/kg BW). None of the piglet reached this point.

### Catheter maintenance and blood collection

Piglets were kept during one week under the sow with their littermates. Piglet behaviour was observed daily to detect signs of prostration or isolation from the sow and littermates. The lumen of catheter was flushed every 48-h with saline solution containing 1% heparin until weaning at d7 to check for catheter functionality. From d7 to d28, one 5-mL blood sample was collected from the catheter in each pig from the Catheter group daily at 8h00. The catheter was flushed with 2 mL of saline solution containing 1% heparin after each blood collection. A blood sample was also collected in the external jugular vein using a 9-mL vacutainer with heparin and a 20G x 25 mm needle in piglets from the Control group on d7, d12, d19 and d26. Blood samples were kept on ice then centrifuged for 10 minutes at 3000 × *g* at 4°C for plasma collection. Plasmas were stored at -20°C until further laboratory analysis.

### Laboratory analysis

Plasma haptoglobin concentration was measured in all collected samples. Haptoglobin concentration in plasma was analyzed using a colorimetric method (Phase Haptoglobin Assay T801; Tridelta Ltd, Maynooth, Ireland) with an automatic analyser (Konelab 20i; Thermo Scientific, France).

### Statistical analysis

Data for BW, DM intake and haptoglobin concentration in plasma were analysed for each day of measurement with piglet as the experimental unit. Data for the average daily gain (ADG) and DM intake were also analysed per period (suckling and post-weaning periods). Statistical analysis was performed using ANOVA for all the parameters using the MIXED procedure of SAS 9.4 (SAS Institute Inc. Cary, NC, USA) including the experimental group as fixed effect and the litter of origin as random effect. The Shapiro–Wilk’s normality test was performed on the residuals for each trait. Differences with a *P*-value under 0.05 are considered significant and with a *P*-value under 0.10 are considered as trend.

Retrospective power calculations were performed for growth and feed intake. With β  =  0.20, α  =  0.05 and standard deviations (SDs) at 49 and 35 g (for ADG and DM intake for the total period respectively), sample size is sufficient to detect a 25% and 13% decrease in ADG and DM intake, respectively, with a *P*-value of 0.05, which were considered to be biologically relevant for a negative response to catheter insertion in piglets (infection, inflammation).

## Results

### Catheterization procedure

One surgeon performed the implantation procedure after familiarisation with the ultrasonography technique (monitoring, ultrasound transducer positioning, and monitored venepuncture). Once the surgeon familiarised with the equipment, catheter implantation using ultrasound guidance was easy to perform. Blood flux colouring with Sonosite® monitor was used during vein localisation and helped avoiding inadvertent arterial puncture. For the seven piglets, time to insert catheter (from anaesthesia induction to catheter fixation) averaged 20 ± 8 minutes (range from 14 to 37 minutes) and anaesthesia duration lasted 27 ± 8 minutes (range from 21 to 42 minutes). Time of separation from the sow (until full recovery including self-mobility) lasted 48 ± 11 minutes (range from 31 to 65 minutes). In all piglets, catheter was inserted in the left external jugular vein (left handler surgeon) at first try.

**Fig 4:**
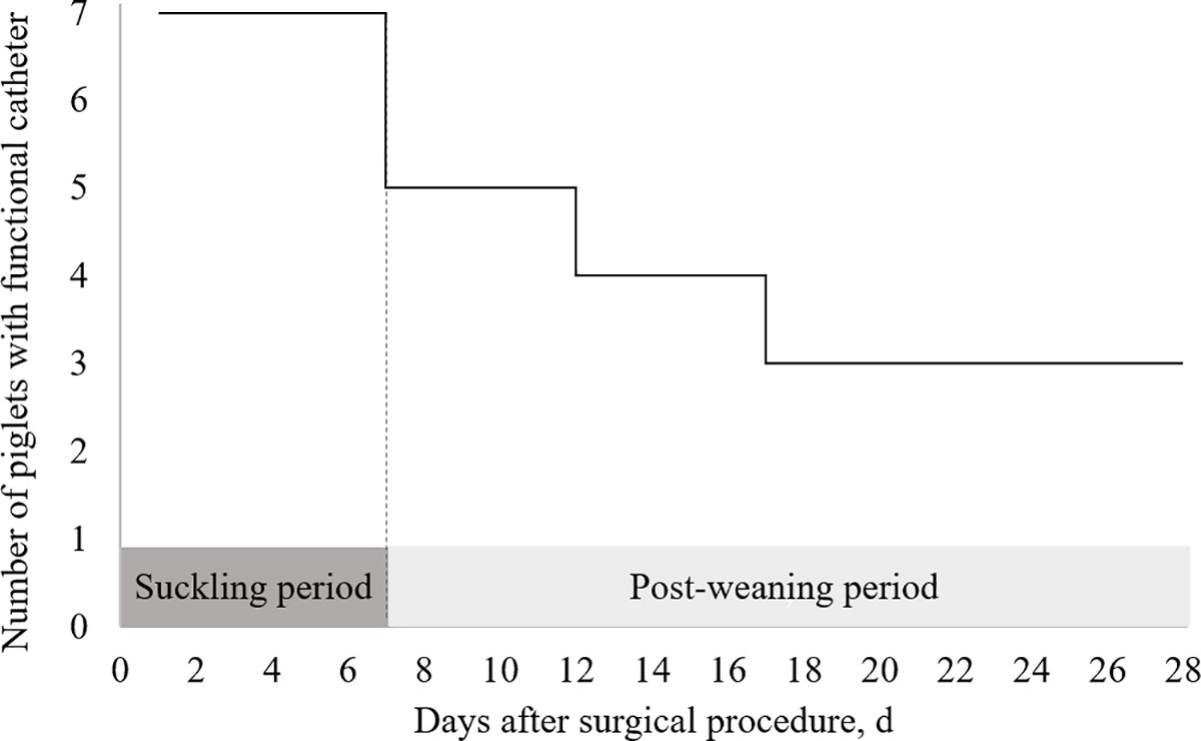
Preservation of catheter function in catheterized piglets (*n* = 7) during the suckling and the post-weaning period.

Piglets tolerated the general anaesthesia and catheter implantation very well. Piglets’ awareness, self-mobility and suckling behaviour were restored within around 15 minutes after anaesthesia was removed. None of the piglets exhibited prostration or stopped suckling during the seven days following the surgical procedure. Over the seven catheters inserted in external jugular vein of piglets at 21 days of age, all catheters remained in place for one week. Catheters were functional for four weeks in three piglets of the seven piglets initially selected for the procedure (Fig 4). Autopsy revealed a thick sheath covering the tip of all non-functional catheters.

### Feed intake

**Fig 5:**
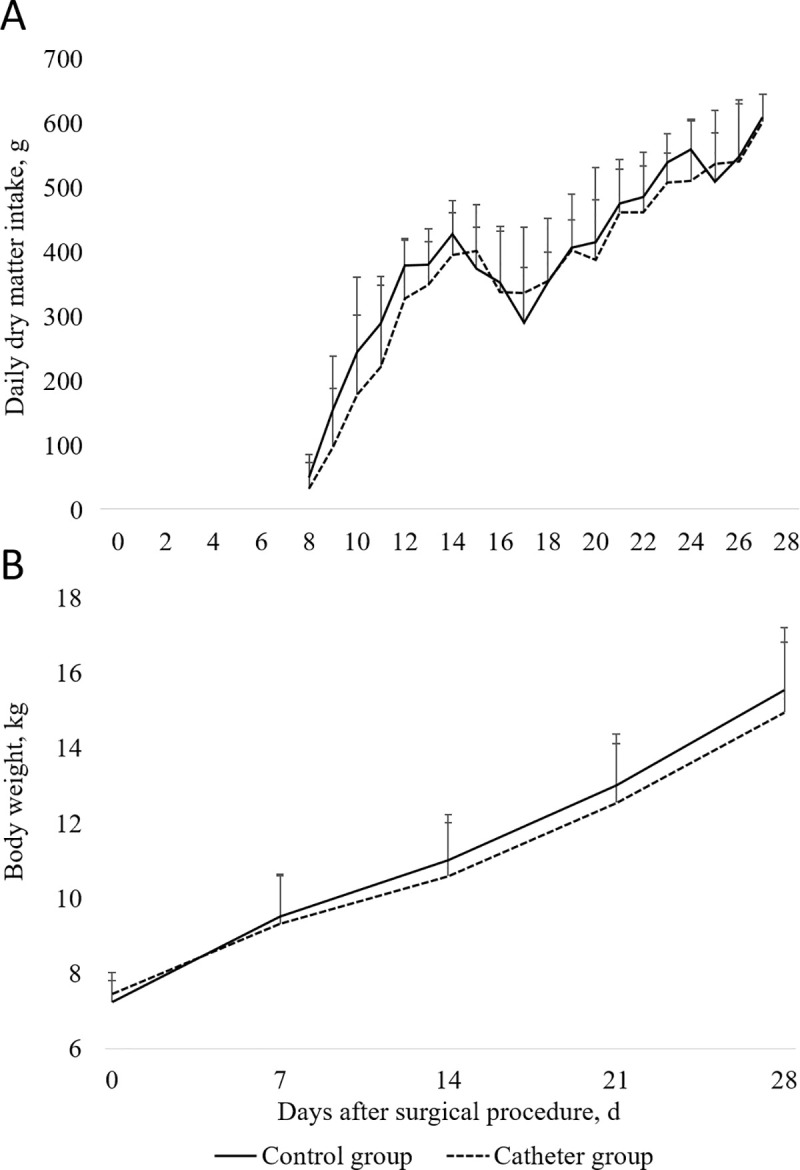
A. Effect of catheter insertion at 21 days of age (d0) on daily dry matter intake of piglets fed *ad libitum* after weaning. B. Effect of catheter insertion at 21 days of age (d0) on body weight of piglets from d0 to d28 after the surgical procedure. Values shown are means ± SDs. Statistical analysis was performed by ANOVA using the experimental group as a fixed effect and the sow as random effect (MIXED procedure, SAS 9.4, SAS Institute Inc. Cary, NC, USA). Dry matter intake did not significantly differ between the Control group (*n* = 7) and the Catheter group (*n* = 7) for each day (*P* > 0,10). Mean body weight did not significantly differ between the Control group (*n* = 7) and the Catheter group (*n* = 7) for each day (*P* > 0,10).

The effect of percutaneous catheterization on feed intake in piglets after weaning is shown on Fig 5A. Daily DM intake increased in both groups during the first week after weaning (d8 to d14 after the surgical procedure), decreased for three days (d15 to d17 after the surgical procedure) and finally increased to reach 606 g DM intake in the end of the experiment. Catheterization did neither affect the DM intake for each day (Fig 5; *P* > 0.10) nor the average calculated from d8 to d14 (230 ± 87 *vs*. 276 ± 45 g DM / day in the Control group; *P* = 0.44), d15 to d17 (359 ± 69 *vs*. 340 ± 61 g DM / day in the Control group; *P* = 0.66), d18 to d27 (477 ± 20 vs. 490 ± 40 g DM / day in the Control group; *P* = 0.46). The averaged DM intake for the total period (d8 to d27 after the surgical procedure) did not differ significantly between the Catheter and the Control group (379 ± 162 *vs*. 393 ± 151 g DM / day; *P* = 0.46).

### Growth

The evolution of BW from d0 to d28 after the surgical procedure is shown on Fig 5. The ADG for the sucking period (d0 to d7) was 324 ± 54 g / day except for one piglet from the Catheter group that exhibited a negative ADG of -53 g / d. From weaning to the end of the experiment, the ADG was 278 ± 47 g / day for all piglets without exception. Catheter insertion did neither affect the BW on d0 (*P* = 0.43), d7 (*P* = 0.38), d14 (*P* = 0.34) and d21 (*P* = 0.40) after the surgical procedure, nor the ADG recorded throughout the study (*P* = 0.18).

### Haptoglobin concentration

**Fig 6:**
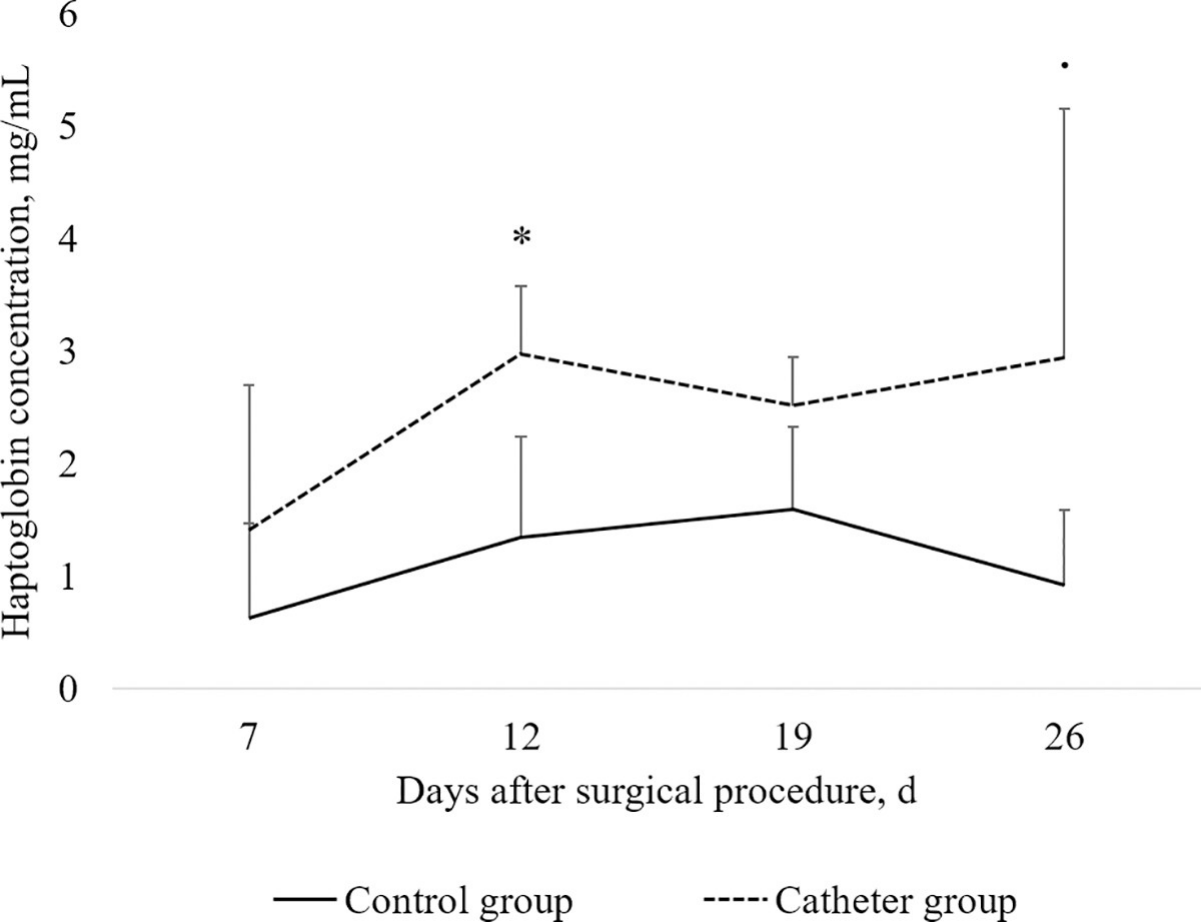
Effect of catheter insertion at 21 days of age (d0) on haptoglobin concentration in plasma of piglets measured at d7, d12, d19, and d26 after the surgical procedure. Values shown are means ± SDs. Statistical analysis was performed by ANOVA for each day using the experimental group as a fixed effect and the sow as random effect (MIXED procedure, SAS 9.4, SAS Institute Inc. Cary, NC, USA). Values that significantly differ (* *P* < 0.05) or tend to differ (. *P* < 0.10) between the Control group (n = 7) and the Catheter group (*n* = 7, 4, 3 and 3 for d7, d12, d19 and d26 respectively) are indicated on the chart.

The effect of percutaneous catheterization on haptoglobin concentration in plasma is shown on Fig 6. Haptoglobin concentration was greater in piglets from the Catheter group than in piglets from the Control group on d12 (*P* = 0.03) and d26 (*P* = 0.08) after the surgical procedure. Haptoglobin concentration did not differ between groups on d0 (*P* = 0.20) and d12 (*P* = 0.14) after the surgical procedure.

## Discussion

### Catheterization procedure

We present a novel method for the percutaneous insertion of an external jugular venous catheter using ultrasound guidance in suckling piglets weighing around seven kilograms. Our aim was to develop a quick, easy and minimally invasive procedure of venous catheterization for repetitive blood collections in piglets around weaning. Percutaneous catheterization minimizes tissue and vessel damage around catheter insertion, in comparison with standard surgical procedures [4, 7–9]. The procedure avoids heavy analgesic protocols, such as the use of opioids [4, 9], that affect animal physiology and behaviour during and after surgery [10]. For experimental purpose, efforts were made in our study to ensure that analgesia does not interfere with inflammatory response to weaning in piglets. According to this, no analgesia, such as nonsteroidal anti-inflammatory drugs (NSAIDs), was administered to animals during the catheterization procedure. Even if continuous measurement or scoring were not performed, no abnormal behaviour associated to pain, such as abnormal posture or gait, social isolation, vocalization, elevated respiratory rate and decreased appetite, were observed in the first hours following the catheterization procedure, or throughout the experiment. However, the administration of NSAIDs or topical lidocaine should be included to the procedure to completely prevent from superficial pain during the procedure when the goal of the experiment is not focused on inflammatory status [11]. Previous descriptions of percutaneous catheterization also report fast recovery after the surgical procedure, thus minimizing the impact of the procedure on feeding and social behaviour of catheterized piglets. In our study, the catheterization procedure was, as expected, fast and greatly reproducible.

Percutaneous catheterization of external jugular vein in pigs according to Seldinger technique has been previously developed [2, 3] sometimes with slight modifications or adds such as table titling 10° head down or the use of a smaller needle [12]. These methods are based on blind venepunctures using landmarks techniques that showed promising results in pigs from 4.8 kg to over 50 kg. However, all methods are difficult to apply, resulting in time-consuming procedures with a low reproducibility (multiple tries needed to succeed in catheter insertion). Furthermore, percutaneous catheterization performed with these procedures may cause inadvertent arterial puncture [2, 3]. Numerous explanations can justify the unease to perform (pig breed, pig positioning during the intervention), the major one being the landmarks subjectivity. Ultrasound guidance has been described as a valuable tool to help reduce time and number of attempts for the placement of central venous catheters in humans [13]. Indeed, ultrasound guidance helps identify and localize the vein and helps orientating the needle for venepuncture. In our study, we used ultrasound guidance throughout the venepuncture procedure to localize the external jugular vein and enter the needle into the lumen. Time for venepuncture was reduced in our study compared to Flournoy et Mani [3] who proposed a similar procedure in small sized piglets using landmarks for vein detection (27.0 vs. 52.5 minutes of total surgical procedure). Furthermore, time range for the procedure was lower compared to the previous method (21 to 42 minutes *vs*. 25 to 120 minutes). Thus, ultrasound guidance successfully helped reduce the time for the surgical procedure and increased reproducibility, mainly when the vein is difficult to find. In our method, we chose a two-step catheterization using a microintroducer set with a small needle (21G) for venepuncture as described by Larsson *et al*. [12] as it is known to limit vessel damage. The use of a 21G needle may have increased precision during venepuncture. A two-step catheterization, using a microintroducer set allowed performing venepuncture using a 21G needle and inserting a 4-Fr catheter to maintain a satisfying blood flux throughout the catheter.

### Catheter functionality

All catheters were kept functional during the first seven days despite piglet’s group housing. From weaning (d7) to the end of the experiment (d28), three of the seven catheters (43%) remained functional. The loss of functionality of catheters after weaning was associated to the development of a sheath over the catheter tip. Catheter-related sheaths are the major cause of catheter dysfunction in pigs [14] and may develop after catheter tip injury to the vein wall [15, 16]. In our study, most of catheter dysfunctions (30%) occurred at weaning step. Social stress in pigs is generally associated with an increase in heart rate [17, 18]. As weaning is associated with an observable increase in physical activity that may rise heart rate during the following hours, weaning may favour catheter tip movement and vessel wall injury. Furthermore, biomaterial that forms along the catheter is known to strongly interact with cells and molecules involved in inflammation [19]. Weaning stressors, especially dietary transition to a solid diet and the loss of immune factors from sow’s milk, are responsible for a transient loss of intestinal integrity and a subsequent increase in systemic immune mediators, such as pro-inflammatory cytokines and acute phase proteins, to cope with intestinal passage of dietary and microbial antigens [20–22]. Taken together, the increased physical activity and the immune response associated with weaning may be taken into consideration as factors that may promote the development of a catheter-related sheath at weaning.

### Effect of catheterization procedure on growth, feed intake and systemic inflammation in piglets around weaning

First aim of the study was to develop a catheter procedure that minimally affects feeding behaviour and growth performance in piglets, especially around weaning. Care was also taken to avoid post-operative drugs including antibiotic treatments, in order to minimally interfere with digestive physiology and health in piglets around weaning that encompass inflammatory response to weaning [21] or changes in intestinal microbiota [23]. Catheter inserted percutaneously did neither affect growth performance in piglets, nor feed intake recorded after weaning. Milk intake could not be measured in our experiment to determine the effect of percutaneous catheterization on feeding behaviour in the first week after the surgical procedure. Milk intake in piglets is correlated to ADG in suckling piglets [24, 25]. Growth was maintained at a similar level in both non-catheterized piglets and catheterized piglets from the first week after the surgical procedure, except for one catheterized piglet. This observation suggests that milk intake may have not been disturbed by the surgical procedure in six over the seven catheterized piglets. The time required for the entire catheterization procedure (48 minutes on average) was less than the average intersuckling interval time [26, 27]. Consequently, catheterization procedure may have minimally affected the frequency of suckling in catheterized piglets, even if the time of suckling was not considered when taking a piglet for surgery. The negative growth observed in one piglet during the suckling period was transient as its feeding level (392 g DM / d) and its growth (256 g / d) during the post-weaning period were similar to those of other piglets. Haptoglobin concentration in plasma for this piglet after the surgical procedure was also within the range of other values measured in this experiment (1.7 mg / mL at d7). This piglet may have expressed a strong and early inflammatory response to catheter with subsequent anorexia. As milk intake and haptoglobin concentration in plasma were not measured during the suckling period, this event may not have been detected.

The increased plasma concentration of haptoglobin in catheterized piglets compared to non-catheterized piglets reveals the induction of a systemic inflammation due to catheter, of significant importance 12 days after the surgical procedure. Because haptoglobin concentration was analysed only for piglets with a functional catheter (*n* = 7, 4, 3 and 3 for d7, d12, d19 and d26 respectively), the increase in haptoglobin content should be evaluated with caution. Nevertheless, it is well known that inflammation provokes a reduction in feed intake and growth [28]. The inflammatory response to catheter seems to be moderate because the elevation of haptoglobin concentration in catheterized piglets was not associated with a reduction in feed intake or growth during the post-weaning period. The inflammatory response to catheter increases within the first two weeks of experiment and may be related to the bacterial colonisation of the catheter. Bacteraemia on the surface of the catheter was not measured in our study. However, the bacterial colonization of the catheter is a common observation in humans or laboratory animals that may lead to septicaemia in few cases [29]. Others also reported the unease to maintain aseptic conditions in unrestrained pigs that promote bacterial colonisation of the catheter [30]. In our study, the catheter was protected with a gauze impregnated with Vetedine solution. Some methodological improvements may help reducing the risk of bacterial colonisation of the catheter and the probably associated inflammatory response, such as the use of a chlorexidin impregnated catheter [31] or a protective chlorexidin gel pad for catheter securement [32]. The suboptimal aseptic conditions for catheter use in unrestrained pigs often leads to complications such as abscesses or fever, even under a prophylactic protocol with antimicrobials administration [30]. In our study, no subsequent general infection in piglets, expressed by a decrease in feed intake, prostration or loss of vitality, was observed. Considering that piglets in our study were group-housed for one week and unrestrained in individual cages until the end of the experiment, the absence of complications, as reported by others [30], indicates that the present procedure can be successfully performed without the use of prophylactic antimicrobials.

## Conclusion

We successfully developed a percutaneous catheterization procedure for piglets weighing seven kilograms using ultrasound guidance. The present procedure shows a great reproducibility and helps avoiding post-operative drugs that may affect animal behaviour and animal physiology. All catheters remained functional during at least seven days. Percutaneous catheterization had a minimal impact on growth and feeding behaviour in piglets within four weeks after the surgical procedure. A moderate inflammation occurred in piglets after the surgical procedure that may be due to vessel wall injury induced by catheter tip movement or bacterial colonisation of the catheter. The present procedure may be transposed to heavier pigs to refine traditional catheterization procedures. Adaptation of the procedure may be needed to cope with differences in anatomy between young and adult pigs.

## Supporting information

S1 Text Time to insert catheter, anaesthesia duration and time of separation from the sow in catheterized piglets. Time to insert catheter (from anaesthesia induction to catheter fixation), anaesthesia duration and time of separation from the sow (expressed in minutes) were recorded during the catheterization procedure in piglets from the Catheter group (n = 7).
(XLSX)

S2 Text Preservation of catheter function in catheterized piglets (n = 7) during the suckling and the post-weaning period. Number of days recorded with functional catheter in piglets from the Catheter group (n = 7).
(XLSX)

S3 Text A. Effect of catheter insertion at 21 days of age (d0) on daily dry matter intake of piglets fed *ad libitum* after weaning. B. Effect of catheter insertion at 21 days of age (d0) on body weight of piglets from d0 to d28 after the surgical procedure.
(XLSX)

S4 Text Effect of catheter insertion at 21 days of age (d0) on haptoglobin concentration in plasma of piglets measured at d7, d12, d19, and d26 after the surgical procedure. (XLSX)
